# Foraging strategies of fungal mycelial networks: responses to quantity and distance of new resources

**DOI:** 10.3389/fcell.2023.1244673

**Published:** 2023-08-24

**Authors:** Yu Fukasawa, Kaho Ishii

**Affiliations:** Laboratory of Forest Ecology, Graduate School of Agricultural Science, Tohoku University, Osaki, Miyagi, Japan

**Keywords:** foraging behaviour, hyphae, migration, soil microcosm, wood decay fungi

## Abstract

Fungal mycelial networks are essential for translocating and storing water, nutrients, and carbon in forest ecosystems. In particular, wood decay fungi form mycelial networks that connect various woody debris on the forest floor. Understanding their foraging strategies is crucial for complehending the role of mycelium in carbon and nutrient cycling in forests. Previous studies have shown that mycelial networks initiate migration from the original woody resource (inoculum) to a new woody resource (bait) if the latter is sufficiently large but not if it is small. However, the impact of energetic costs during foraging, such as the distance to the bait, has not been considered. In the present study, we conducted full-factorial experiments with two factors, bait size (4 and 8 cm^3^) and distance from the inoculum (1 and 15 cm). An inoculum wood block, colonized by the wood decay fungus *Phanerochaete velutina*, was placed in one corner of a bioassay dish (24 cm × 24 cm) filled with unsterilized soil. Once the mycelium grew onto the soil to a distance >15 cm from the inoculum, a sterilized new bait wood block (of either size) was placed on the soil at one of the two distances to be colonized by the mycelia from the inoculum. After 50 days of incubation, the baits were harvested, and their dried weight was measured to calculate the absolute weight loss during incubation. The inoculum wood blocks were retrieved and placed on a new soil dish to determine whether the mycelium would grow out onto the soil again. If no growth occurred within 8 days of additional incubation, we concluded that the mycelium had migrated from the inoculum to the bait. The results showed that mycelia in inocula coupled with baits positioned 1 cm away migrated to the baits more frequently than those with baits positioned 15 cm away. A structural equation model revealed that bait weight loss (energy gain) and hyphal coverage on the soil (foraging cost) significantly influenced mycelial migration decisions. These findings suggest that fungal mycelia may employ their own foraging strategies based on energetic benefits.

## 1 Introduction

Mycelial networks of fungi, which extend the litter-soil interface on the forest floor, play important roles in the forest ecosystem. In the case of decomposer fungi, mycelial networks connect numerous units of dead wood and leaf litter, contribute to the decomposition and mineralization of those plant tissues (Boddy, 1999). In the case of mycorrhizal fungi, mycelial networks connect symbiotic tree roots, forming complex, interwoven common mycorrhizal networks (Simard et al., 1997). In both cases, mycelial networks facilitate the transfer of carbon (Wells et al., 1995; Simard et al., 1997), nutrients (Wells et al., 1990; Kiers et al., 2011), and even information (Johnson and Gilbert, 2015) across their expansive bodies, which can cover hundreds of hectares (Ferguson et al., 2003). Gaining a better understanding of the factors that affect the development of mycelial networks is crucial for predicting dynamics of forest ecosystems in a changing environment.

Resources for wood decay fungi are dead wood, which is unevenly distributed on the forest floor. Therefore, strategies for foraging for these dead woods, including which ones to colonize preferentially and when to leave old ones for new resources, are important for the optimal management not only of energy gain and survey cost but also of network structure, which is vital for efficient material transfer across mycelial networks (Fricker et al., 2017). Previous reports have shown that flexible behaviour of mycelial networks depends on the quantity and timing of resource addition, shedding light on their economic strategies. For example, mycelia of *Phanerochaete velutina*, a known basidiomycete species that forms mycelial “cords” (visible strand made of numerous parallel-running hyphae), completely leave old wood blocks after colonizing sufficiently large new wood blocks but do not abandon old ones if a new resource is small or the old ones are still nutritious (Fukasawa et al., 2020; Fukasawa and Kaga, 2021). This species also exhibits adaptive behaviour in response to grazing pressure from soil invertebrates by developing more highly-connected robust networks (Boddy et al., 2010), as well as in response to repeated artificial disturbances based on memory to minimize physical damage (Donnelly and Boddy, 1998). These results suggest that mycelial networks can also manage risk and cost when developing their networks. However, how a mycelium behaves when they faced with multiple issues simultaneously remains unclear. For example, what choices do they make when they encounter a large new resource (with significant benefits) far from their original location (involving substantial foraging costs), compared to a small new resource located much closer? Given the uneven and patchy distribution of dead wood on the forest floor, such situations are likely to be quite common. Nevertheless, few studies have explored fungal behaviour in multiple tasks.

In the field of behavioural ecology, which has mostly been developed to explain animal behaviour, optimal foraging strategies (OFS) are known to maximize net energetic gain by subtracting the costs associated with foraging activities (Davies et al., 2012). For individual motile organisms with clear individuality (e.g., animals), the time consumed for foraging is one of the most important factors determining the foraging cost (Krebs, 1973). On the other hand, for modular organisms such as plants, slime moulds, and fungi, which can flexibly enlarge their body size and shape but usually sessile, the distance (or spatial scale) to the resource could be an important factor determining the cost of their foraging activities (Dussutour et al., 2019; Zheng et al., 2022). Slime mould *Physarum polycephalum*, in particular, has been extensively studied for its ability to find the shortest and least stressful paths connecting multiple resources (Nakagaki et al., 2000a; Tero et al., 2010) and for its decision-making process on when to leave old resources (Latty and Beekman, 2009, 2015). Mathematical models have even been proposed to explain their adaptive behaviours (Tero et al., 2007; Lecheval et al., 2021; Hu et al., 2022). The Marginal Value Theorem (MVT) predicts that the optimal strategy for foragers is to leave patches when the instantaneous rate of return in the patch falls to the average of the returns that can be achieved in all the other patches within the environment (Latty and Beekman, 2015).

**FIGURE 1:**
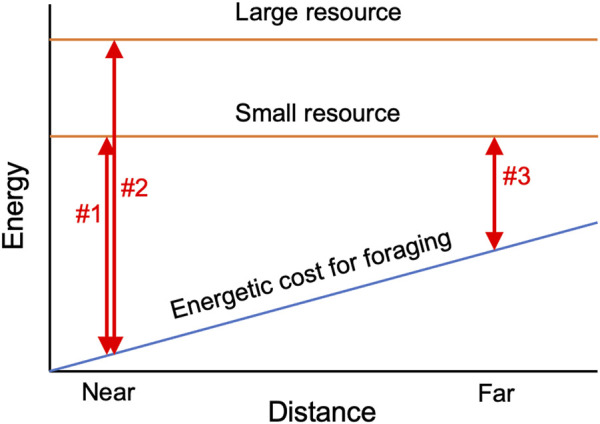
Schematic diagram explaining the hypothesis tested in the present study. Both the size and distance of the resource may impact the net energy gain (double-head arrows in the figure) by influencing the energetic cost of foraging. If the distance to the resources are equal, net energy gain might be mainly restricted by the size of the resource (comparison between arrow#1 and arrow#2), whereas if the distance to the resources are different, energetic cost for foraging might affect the difference in the net energy gain (comparison between arrow#1 and arrow#3).

In contrast to slime moulds, the behavioural ecology of fungi is still in its infancy (Money, 2021), and their OFS has not been well explored in terms of balancing costs and benefits. Since hyphal production required to reach new resources is an energetic cost for fungi, finding a new resource located far from the original inoculum might be energetically more expensive than finding closer resources. Thus, a mycelium might be expected to obtain a larger net energy from closer resources compared to resources located far from the inoculum, assuming the size (or energetic value) of the new resources is equal (Figure 1). In fact, the size of the resource is important for mycelial decision-making, as mentioned above (Fukasawa et al., 2020), likely reflecting the difference in the amount of energy available from these resources. Regarding distance, we hypothesize that a mycelium will leave the inoculum and migrate to a new resource more frequently when the new resource is located closer to the inoculum, as the net energy gain from the new resource might be larger when it is closer (Figure 1).

The aim of this study was to evaluate the effects of the size and distance of new wood resources (baits) on the decision-making process of a fungal mycelium to migrate from an old inoculum to the bait. We hypothesized that larger and closer baits would induce more migration of the mycelium than smaller and farther baits because a mycelium can obtain more energy from the former set of baits compared to the latter set of baits per unit foraging effort, after subtracting the energetic cost. We used a soil dish microcosm and a saprotrophic cord-forming basidiomycete, *Phanerochaete velutina* (DC.) P. Karst., as a model system. This fungus is one of the most well-studied species in the research field of mycelial network behaviour (Boddy, 2009; Fukasawa et al., 2020; Fukasawa and Kaga, 2021). Absolute weight losses of the wood blocks (inoculum and bait) were measured as indices of energy gain, while hyphal coverage on the soil dish was measured as an index of the energetic cost for the mycelium during the foraging operation.

## 2 Materials and methods

### 2.1 Fungal culturing and inoculum preparation

Beech (*Fagus crenata*) wood was cut into blocks measuring 0.5 cm × 1 cm × 1 cm (0.5 cm^3^) and dried at 70°C until the constant weight was achieved. The numbered blocks were soaked overnight in distilled water and then autoclaved at 121°C for 20 min. The autoclaving process was repeated three times with 1 day intervals to ensure sterilization. The sterilized wood blocks were placed on cultures of *P. velutina* (NBRC culture collection, #110184) grown on 0.5% malt extract agar (MEA; 5 g Lab M malt extract, 15 g Lab M agar no. 2) in non-vented 9 cm-diameter Petri dishes (1.5 cm thick). The plates were sealed with Parafilm^®^ (Bemis Company Inc., Oshkosh, United States), and incubated in the dark at 20°C for 1 month before use. In total, 113 inoculum wood blocks were prepared in 11 Petri dishes, with 10–11 blocks in each dish. The whole experimental flow is shown in Supplementary Figure S1.

### 2.2 Microcosm preparation

Soil was collected from the top 10 cm (A layer) of a deciduous mixed forest dominated by *Quercus serrata* and *Larix kaempferi* in Kawatabi Field Science Center of Tohoku University, Miyagi, Japan (38°45′ N, 140°45′ E, 275 m a.s.l.). The soil was sieved on site using a 10 mm mesh, air-dried, sieved again through a 2 mm mesh, and frozen at −30°C for more than 48 h to kill soil invertebrates. The soil was then rehydrated with distilled water (70 mL for 130 g dried soil), transferred to 24 cm × 24 cm plastic bioassay dishes, smoothed and compacted to a depth of approximately 5 mm (approximately 200 g wet soil for each dish).

An inoculum wood block, from which surface mycelia and excess agar had been removed using a razor blade, was placed 1 cm from a corner of each dish. All dishes were incubated at 20°C in the dark in BioTRON (NK system, Osaka, Japan) for 50 days. This period is referred to as the pre-incubation period. Out of the total of 113 soil dishes with inoculum wood blocks, 40 dishes where mycelia had extended more than 15 cm from the inoculum wood block were selected for the downstream experiments. A new beech wood block (bait), prepared and sterilized as described above, was placed on each of the 40 dishes at two different distances from the inoculum: one at 1 cm (NEAR experiment) and the other at 15 cm (FAR experiment) away from the inoculum. Two sizes of bait wood blocks were used: 2 cm × 2 cm × 1 cm (4 cm^3^, SMALL) and 2 cm × 4 cm × 1 cm (8 cm^3^, LARGE). Thus, a total of four experiments were conducted (two distances × two bait sizes), with ten replicates for each experiment. The 73 inoculum wood blocks that were not used in the further incubation were harvested and dried at 70°C to a constant weight. The weight loss (%) of the inoculum wood blocks after the pre-incubation period was calculated using the following equation:

$$Weight\;loss\;\left(\%\right)=\frac{original\;dried\;weight-dried\;weight\;after\;preincubation\;period}{original\;dried\;weight}\times 100$$

The weight loss (%) data were used to create a regression line between weight loss (%) and hyphal coverage (cm^2^) on the soil, which was measured as described later. Given the linear correlation between wood weight loss and hyphal coverage on soil (Fukasawa and Kaga, 2020), this regression line was used to estimate the weight loss (%) of the 40 inocula used for the further incubation experiment at the end of the pre-incubation period.

### 2.3 Microcosm incubation

All dishes were weighed after set-up, and their weight was monitored weekly to replace lost water by spraying distilled water evenly across the soil surface. The dishes were stacked in polythene bags to reduce water loss and were incubated at 20°C in the dark for 50 days in BioTRON (period I).

After period I, bait blocks were harvested, and their surface mycelia and soil were removed using a razor blade. The blocks were then dried at 70°C until a constant weight was achieved. Inoculum wood blocks were retrieved, scraped-off mycelia and soil on their surface, and placed centrally onto new soil dishes freshly prepared as described above but in smaller round dishes with a diameter of 14 cm and a thickness of 2.5 cm. In cases where the inoculum wood blocks were too soft due to fungal decay activities, the soil under the blocks was cut out together with the blocks and transferred to the new dishes. The dishes were further incubated at 20°C in the dark for 8 days to check for mycelial regrowth from the inoculum (period II). If mycelial regrowth was not observed, it was recorded as migration from the inoculum to the bait.

During incubation period I, the dishes were randomly repositioned every 3 days to avoid possible effects of orientation and location within BioTRON on the direction of hyphal growth. After period II, the inoculum wood blocks were harvested, cleaned of surface mycelia and soil, and dried at 70°C to constant weight. The absolute weight loss (g) of the inoculum and bait wood blocks was calculated by subtracting the dried weight after the incubation period from the original dried weight of each wood block. We used the absolute weight loss of the wood blocks in the analyses as a proxy for the energy obtained by *P. velutina* from the wood blocks because the absolute weight loss is different between resource sizes even if the percentage weight loss is equal (Fukasawa and Kaga, 2020).

### 2.4 Image analysis

The dishes were photographed at the end of the pre-incubation period, every 3 days during incubation period I, and at the end of period II, using Canon EOS Kiss X10 camera, equipped with Canon EF-S18-55 mm F4-5.6 IS STM lens, mounted on a stand at a height of 52 cm, under the same light conditions to ensure consistency. The photo images at 24th day of period I were used to judge whether the mycelium colonized to the bait wood block from the inoculum side or from the opposite side (Supplementary Figure S2). The photo images at the end of period I were analyzed using ImageJ (National Institute of Health, United States) to evaluate the hyphal coverage on soil. The length of one side of the inoculum wood block (1 cm) was used as a calibration ruler. The edges of each soil dish and the wood blocks were removed by windowing, and the resulting images were converted to black (mycelia) and white (soil) using a manually set threshold. Hyphal coverage (cm^2^) on the soil was used as a measure of hyphal biomass, representing the cost for *P. velutina*.

### 2.5 Statistical analysis

All analyses were performed using R 4.2.2 (R core team 2022). The normality of data distribution in each experiment was tested by Shapiro-Wilk test and equality of data variance across the experiments was tested by Bartlett test. Absolute weight losses of inoculum and bait were compared among the four experiments by Tukey-Kramer test because the data have normal distribution and equal variance across the experiments, whereas non-parametric Steel-Dwass test was employed for hyphal coverage at the end of period I due to lack of normality in one of the experiments. The frequency of mycelial migration from the inoculum to the bait wood blocks was compared between the two distance settings within experiments using same size of bait, employing Fisher’s exact test.

The effects of bait size and the distance between the inoculum and bait on the absence of hyphal regrowth in period II (i.e., migration) were evaluated using a generalized linear model (GLM). A binomial distribution error was assumed, and a logit link function was used. The model did not include an interaction term of bait size and distance because our hypothetical scenario (Figure 1) predicts an interactive effect of resource size and distance on hyphal behaviour only when the distance is sufficiently large to make the cost for hyphal production larger than the energy available from resource. In this study, the hyphal production cost needed for resource survey might be much smaller than the energy available from the wood blocks, even in the FAR experiment, as hyphae grew out from the inoculum at one corner of the dish and reached the opposite side of the dish in most cases.

The indirect effects of bait size and distance on the absence of hyphal regrowth in period II (i.e., migration) through the weight losses of inoculum and bait wood blocks (energy benefit) and hyphal coverage (energy cost) were evaluated using structural equation modeling (SEM) with the lavaan package version 0.6-13 (Rosseel et al., 2023). Bait size and distance were set as the first-order variable, and the absolute weight losses of inoculum and bait wood blocks and hyphal coverage at the end of period I were set as the second-order variables. Link arrows pointed from lower- to higher-order variables, but the following links were removed from the model due to a lack of rationality: from bait size to inoculum weight loss, from distance to inoculum weight loss, and from distance to bait weight loss.

## 3 Results

**FIGURE 2:**
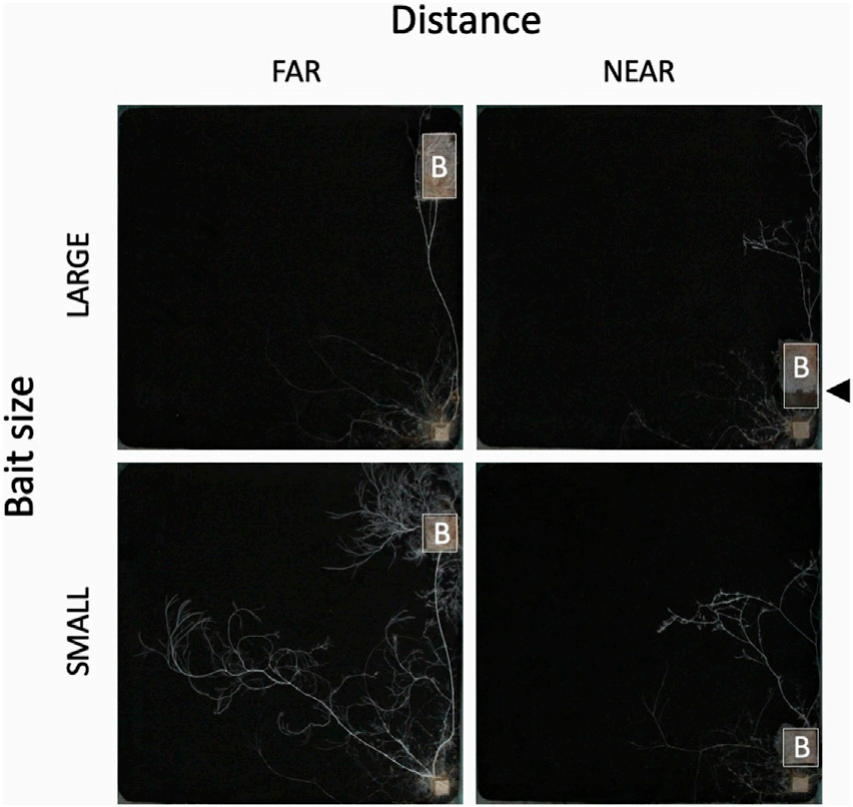
The effect of bait (B) size and distance on the development of *Phanerochaete velutina* mycelial systems originating from an inoculum wood block (small block at the bottom right corner). All photos were taken at the end of incubation period I. Bait size: 4 and 8 cm^3^ for SMALL and LARGE, respectively. Bait distance: 15 and 1 cm for FAR and NEAR, respectively. The outline of the bait wood block is delineated by a white line for clarity. Note that in the LARGE-NEAR experiment (upper-right photo), mycelium colonization on the bait occurred from the side opposite the inoculum. Black arrowhead indicates the colonization front line of mycelium colonized from the side opposite to the inoculum.

**FIGURE 3:**
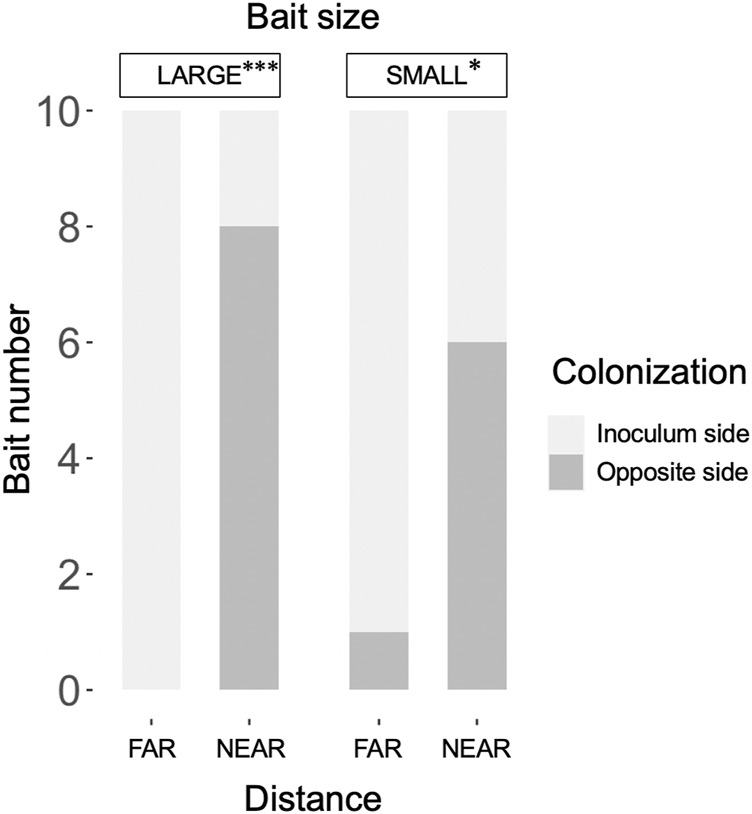
Frequency of hyphal colonization in LARGE and SMALL bait wood blocks, positioned at different distances from the inocula, from the side opposite the inoculum. The frequency was compared between the FAR and NEAR experiments (Fisher’s exact test: *, *p <* 0.05; ***, *p <* 0.001).

**FIGURE 4:**
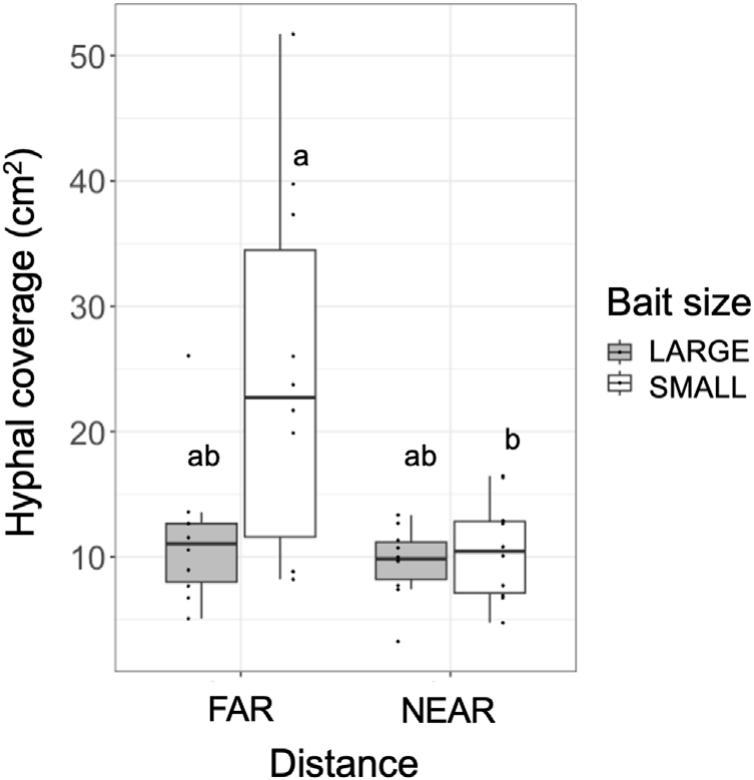
Hyphal coverage (cm^2^) of mycelia extending from the inoculum wood block onto the soil at the end of incubation period I. Different letters on each box indicate significant (*p* < 0.05) differences among the four combinations of distance and bait (Steel-Dwass test, *N* = 10).

The weight loss of the 73 unused (in incubation periods I and II) inoculum wood blocks at the end of the preincubation period was on average (± standard error) 48.0% (±0.8%; range: 22.4%–60.9%). For the 40 used (in incubation periods I and II) inoculum wood blocks, the estimated weight loss at the end of the preincubation period (start of incubation period I) was on average 53.0% (±0.42%; range: 45.2%–58.2%), based on the regression line between weight loss and hyphal coverage (Supplementary Figure S3). Hyphae, grew out from the 40 inoculum wood blocks, successfully colonized on the baits regardless of their size and distance from inocula during incubation period I (Figure 2). Interestingly, in the FAR experiments, hyphal colonization on the bait started from the side facing the inoculum, whereas in the NEAR experiments, it frequently started from the opposite side of the bait (black arrowhead in Figures 2, 3). Hyphal coverage at the end of period I appeared to be larger in FAR compared to NEAR experiment in SMALL bait (Figure 4, Steel-Dwass test, *p* = 0.04, between FAR-SMALL and NEAR-SMALL experiments).

**FIGURE 5:**
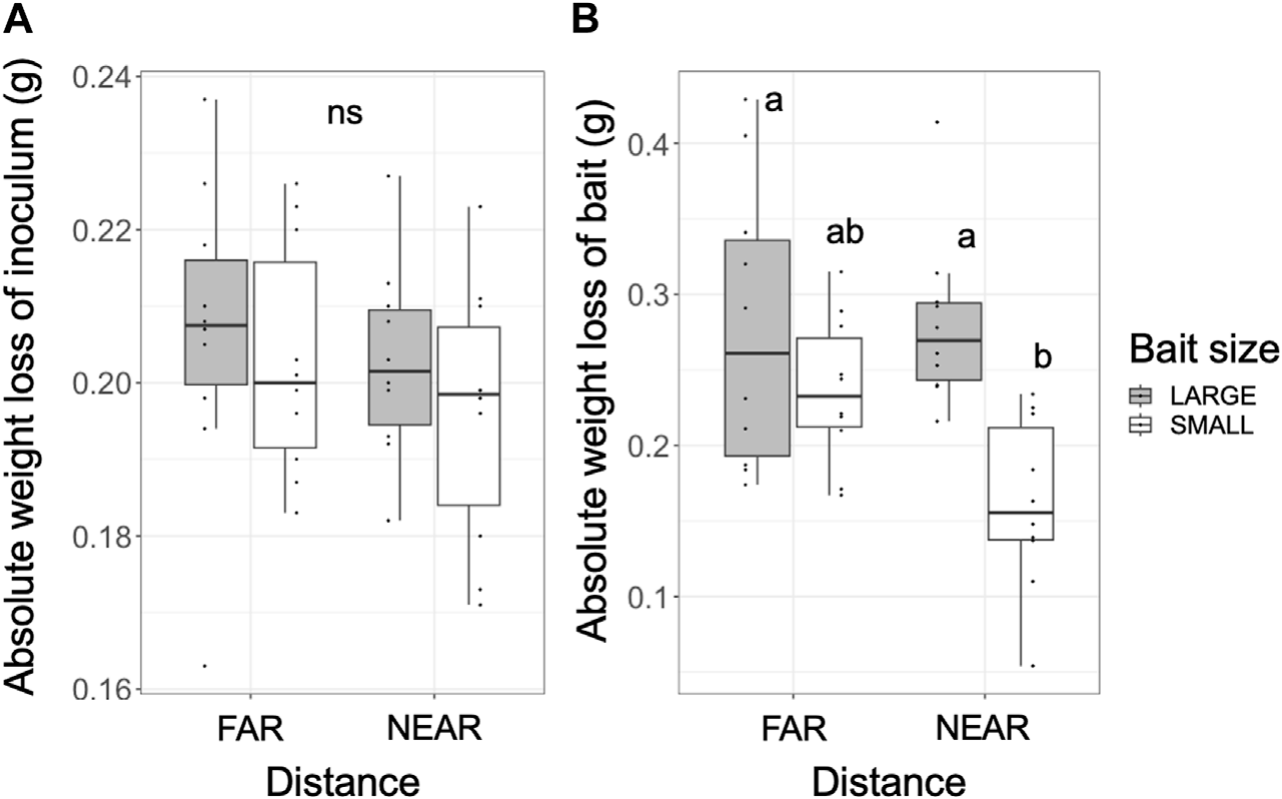
Absolute weight loss of the inoculum **(A)** after incubation period II and the bait **(B)** after incubation period I. Different letters on each box indicate significant (*p* < 0.05) differences among the four combinations of distance and bait (Tukey-Kramer, *N* = 10). ns, not significant.

The absolute weight loss of the inoculum after the experiment (period II) was 0.163–0.237 g, representing 65%–92% of the original dried weight, and there were no significant differences across the experiments (Figure 5A). However, the absolute weight loss of the bait after period I was 0.054–0.429 g, representing 2.5%–14.2% of the original dried weight (Figure 5B). The weight loss of the bait was significantly lower in the NEAR-SMALL experiments compared with the other experiments (Figure 5B).

**FIGURE 6:**
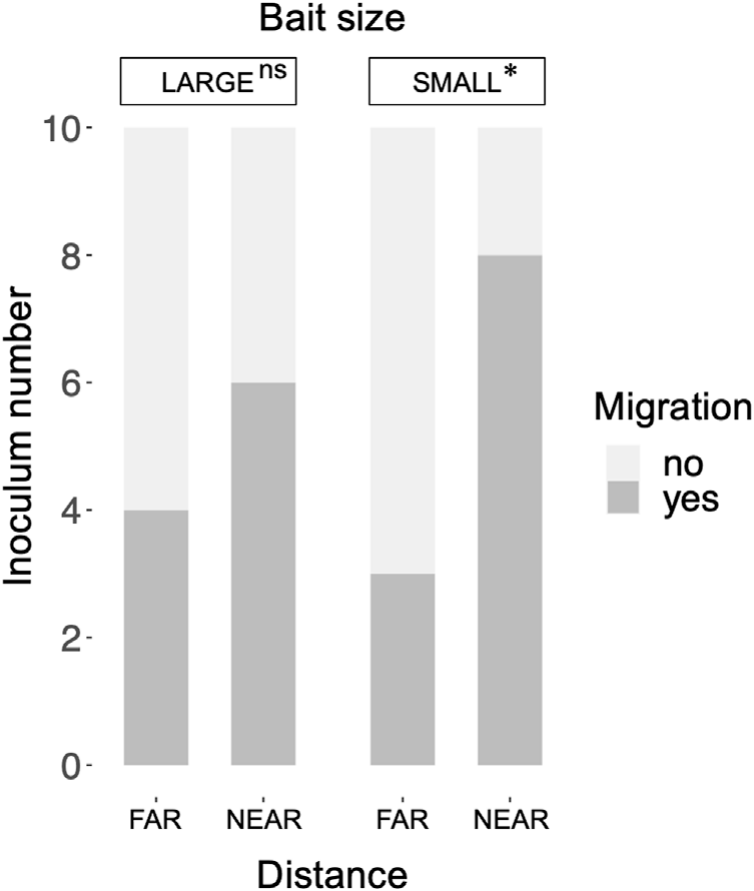
Frequency of mycelium migration in LARGE and SMALL bait wood blocks positioned at different distances from the inocula. Migration frequency was compared between the FAR and NEAR experiments (Fisher’s exact test: *, *p* < 0.05; ns, not significant).

Mycelial migration from the inoculum to the bait (i.e., no regrowth from the inoculum in incubation period II) was observed more frequently in the NEAR experiments (8/10) than in the FAR experiments (3/10) with SMALL bait (Figure 6). However, there was no significant difference in migration frequency between the NEAR (6/10) and FAR (4/10) experiments with LARGE bait (Fisher’s exact test, *p* = 0.33).

The results of the GLM indicated that the distance between the inoculum and bait significantly influenced migration occurrence (Table 1), with migration occurring more frequently when the distance was shorter. However the effect of bait size was not significant.

**TABLE 1:** GLM results explaining absence of hyphal regrowth in period II (i.g., migration) of mycelium from inoculum to bait wood block.

Factor	Estimate	S.E.	*Z* value	*p*-value
Bait size (SMALL)	0.229	0.678	0.338	0.7355
Distance (NEAR)	1.471	0.678	2.169	0.030

**FIGURE 7:**
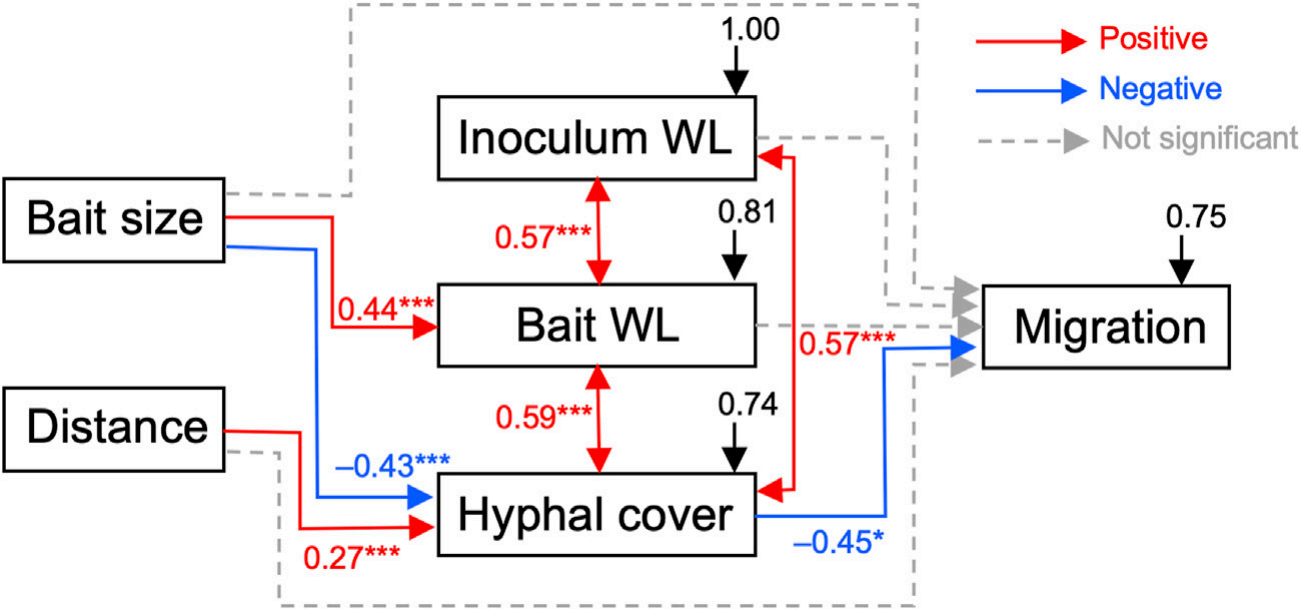
Results of structural equation modeling (SEM) showing potential causal relationships between bait size and distance, absolute weight losses of the inoculum and bait, hyphal coverage on the soil dish, and the presence of mycelial migration (*p*-value of Chi-square test = 0.262; comparative fit index [CFI] = 0.984; root mean square error of approximation [RMSEA] = 0.091; standardized root mean square residual [SRMR] = 0.09). Arrows represent causal pathways: red, blue, and dotted gray arrows indicate positive pathways, negative pathways, and non-significant pathways, respectively. *, *p* < 0.05; ***, *p* < 0.001. Numbers beside arrows represent path coefficients, taking values between −1 and 1, and difference from 0 indicates the strength of the relationship. Black arrows represent residual errors that arise in the prediction of the effects of unmeasured factors to the measured variables.

Structural equation modeling (SEM) analysis results revealed positive correlations between inoculum and bait weight losses and hyphal coverage (Figure 7). *p*-value of chi-square test > 0.05, comparative fit index (CFI) > 0.9, and root mean square error of approximation (RMSEA) < 0.1, indicated good model fit of the data. Additionally, bait weight loss was positively correlated with bait size. Hyphal coverage was positively correlated with the distance between the inoculum and bait, but negatively correlated with bait size. The occurrence of mycelial migration was negatively correlated with hyphal coverage. However, the direct effects of the factors, excluding hyphal coverage, on migration occurrence were not significant.

## 4 Discussion

SEM analysis results showed that when the bait was located far from the inoculum, there was an increase in hyphal coverage on the soil, which corresponded to a lower frequency of mycelial migration from the inoculum to the bait (Table 1 and Figure 7). This initial finding appears to support our hypothesis. However, we must exercise caution in discussing whether this is a result of high foraging costs associated with maintaining hyphae on the soil. Upon examining Figure 2, it seems that the increase in hyphal coverage in the FAR-SMALL experiment was not due to long connecting hyphae between the inoculum and bait but rather the secondary growth of foraging hyphae from the bait. This observation is further supported by the data from the LARGE experiments, where secondary growth of foraging hyphae was not observed, nor was there a difference in hyphal coverage between the NEAR-LARGE and FAR-LARGE experiments (Figure 4). Therefore, in the present study, the difference between the 1 and 15 cm distances from the inoculum to the bait may not have significantly affected the cost to maintaining hyphal connections between the inoculum and bait wood blocks. Instead, in the FAR-SMALL experiment, hyphal growth was also activated in a different direction without the presence of bait, suggesting that the inoculum serves as a hub for the established hyphal network (Figure 2). Conversely, such activation of hyphal growth in the direction without bait was not observed in other experiments. The reason for this difference becomes evident when examining the images of the NEAR experiment, shown in Figures 2, 3, where it is clear that hyphal colonization of the bait occurred from the side opposite the inoculum. In the present study, the hyphae of *P. velutina* were allowed to grow more than 15 cm from the inoculum before baiting, even in the NEAR experiment, to maintain uniform experimental conditions (excluding bait size and distance). Thus, in the NEAR experiment, the bait was placed on the basal part of the hyphal cord elongated from the inoculum. Therefore, the finding that hyphal colonization of the bait occurred from the side opposite the inoculum suggests that the main cytoplasmic body of the hyphae was located at its growing front, rather than in close proximity to the inoculum at the time of baiting.

Hyphae grow at their tips, where they exhibit strong directionality (Held et al., 2019). Particularly in a young growing colony, cytoplasmic flow, including nutrients, is concentrated toward the growing front, resulting in a larger nutrient content at this front than compared with the center of the colony (Tlalka et al., 2007). When a new bait is placed in close proximity to the inoculum (center) of such a growing colony, the majority of cytoplasm directed toward the growing front must reverse its flow to colonize the bait located at the center. This could explain why colonization of hyphae on large bait occurred from the side opposite the inoculum. For the same reason, colonization of NEAR baits may have taken more time than colonization of FAR baits, thereby delaying decomposition, especially on SMALL bait [although not statistically significant (Figure 5)]. These results suggest that the energy gain from bait may be greater in FAR experiments than in NEAR experiments. This contradicts our assumption that the net energy gain from a new resource would be larger when it is located closer.

If the migration of mycelium cannot be explained solely by the net energy gain from a new resource, an alternative explanation must be found. The foraging and migration behaviour of slime mould plasmodia have been studied extensively. Though unicellular, those organisms, have a superficially similar body design to fungal mycelia (Westerhoff et al., 2014; Boussard et al., 2021). Numerous studies on the model species *Physarum polycephalum* have revealed that plasmodia can optimize their network structure, connecting separately located multiple resources, by adjusting local cytoplasmic flow in response to the location, quantity, and quality of resources (Nakagaki et al., 2000b; Nakagaki and Guy, 2008). Similarly, previous studies on material transport within the mycelial network of *Phanerochaete velutina* have reported that nutrients, such as phosphorus, are transferred from the inoculum to bait wood blocks in relation to the size and quality of the bait (Fricker et al., 2017; Fukasawa et al., 2020). As demonstrated in the present SEM analysis, large bait reduced hyphal coverage on the soil and induced mycelial migration towards the bait. Furthermore, the migration decision of *P. velutina* mycelium was affected by the waiting time for the bait, i.e., the quality change of the original inoculum wood (Fukasawa and Kaga, 2021). If mycelium, as a modular organism, responds locally to these environmental stimuli (Itani et al., 2023), the NEAR experiment in the present study may have resulted in two separate parts of the mycelium responding to the bait, more or less, independently: the small part of the mycelium close to the original inoculum, and the larger spreading fans, including the growing front. Given that most of the nutrients are likely directed toward the growing front, the small part of the mycelium must respond to the new bait without a sufficient allocation of nutrients, which likely induces migration. Therefore, the difference in migration frequency observed in the present study could be attributed to the position of the new bait within a mycelial network that inherent possesses heterogeneity in its cytoplasm and nutrient distribution, rather than simply the distance between the inoculum and bait. A wood block on the soil (comprising both the inoculum and bait) serves as a carbon source for fungi, but it also functions as a base from which the fungi extend their hyphae into the soil to absorb nutrients. Hence, it might be more reasonable to position an old inoculum (a low-quality carbon source) far from the new bait, serving as a base (i.e., without migration), to facilitate the spread of hyphae into a larger area of soil, rather than keeping the old inoculum close to the new bait.

In the present study, we allowed the hyphae to grow more than 15 cm from the inoculum in all experiments before baiting to standardize the baiting time across the experiments. This resulted in a difference in the baiting position within the mycelium between the FAR and NEAR experiments. Bait was placed at the growing front of the hyphae in the FAR experiments, but it was positioned on the hyphal cord, far from the growing front, in the NEAR experiment. The initial hyphal elongation in both experiments may have reduced the difference in foraging costs between the FAR and NEAR experiments. To amplify the cost difference, an experimental approach could involve placing baits at the growing front of the mycelia in both FAR and NEAR experiments. However, this would introduce a difference in baiting time between the experiments, as hyphae takes 50 days to grow up to 15 cm. This difference in baiting time could potentially impact the quality of the inoculum and the nutrient status of the mycelium. On average, a 53% weight loss was estimated in the inocula during 50 days on the soil, with this result based on the regression line of 73 additional inocula not used in the baiting experiment. Overall, simultaneously unifying bait timing and distance from the inoculum across all experiments remains challenging.

The results of the present study reveal that the migration behaviour of *P. velutina* mycelia is influenced by bait size and its distance from the inoculum. Although the effect of bait size was not clear compared to previous study (Fukasawa et al., 2020), it might be attributable to the small difference in bait size in the present study. The results also suggest that the position of the bait within the mycelial network may play a crucial role in mycelial behaviour, rather than the distance between the inoculum and bait. To gain a deeper understanding of the foraging strategy of fungal mycelium, further microcosm experiments are warranted, focusing on mycelial behaviour in relation to the heterogeneity of resource utilization within a mycelium, particularly in systems involving multiple wood resources.

## Data Availability

The raw data supporting the conclusion of this article will be made available by the authors, without undue reservation.
