# Supplementary material for: Foraging strategies of fungal mycelial networks: responses to quantity and distance of new resources
Source: Front Cell Dev Biol. 2023 Aug 24;11:1244673. doi: 10.3389/fcell.2023.1244673 (PMC10483288; doi:10.3389/fcell.2023.1244673)
Supplement: Supplementary file 1 [file DataSheet1.PDF]

Supplementary Material

Foraging strategies of fungal mycelial networks: responses to quantity and distance of new resources

Yu Fukasawa*, Kaho Ishii

*** Correspondence:** Yu Fukasawa: yu.fukasawa.d3@tohoku.ac.jp

1 Supplementary Figure 1

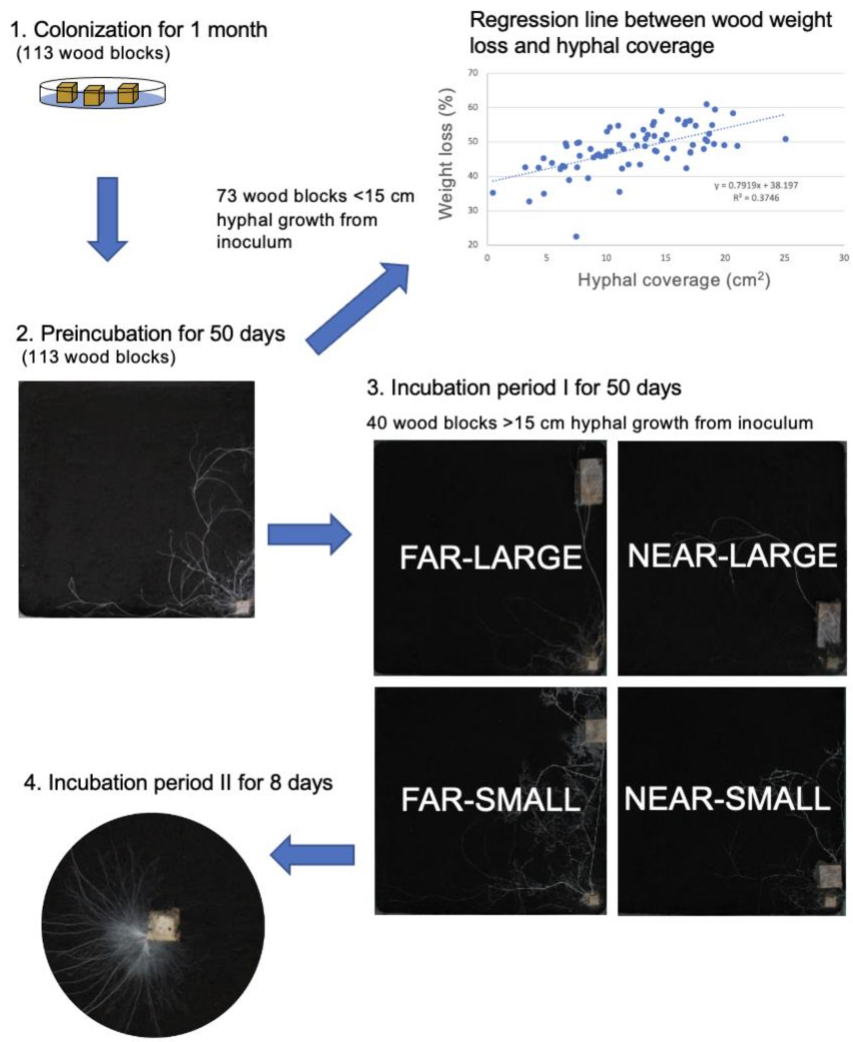

Supplementary Figure 1. Schema of the experimental flow.

2 Supplementary Figure 2

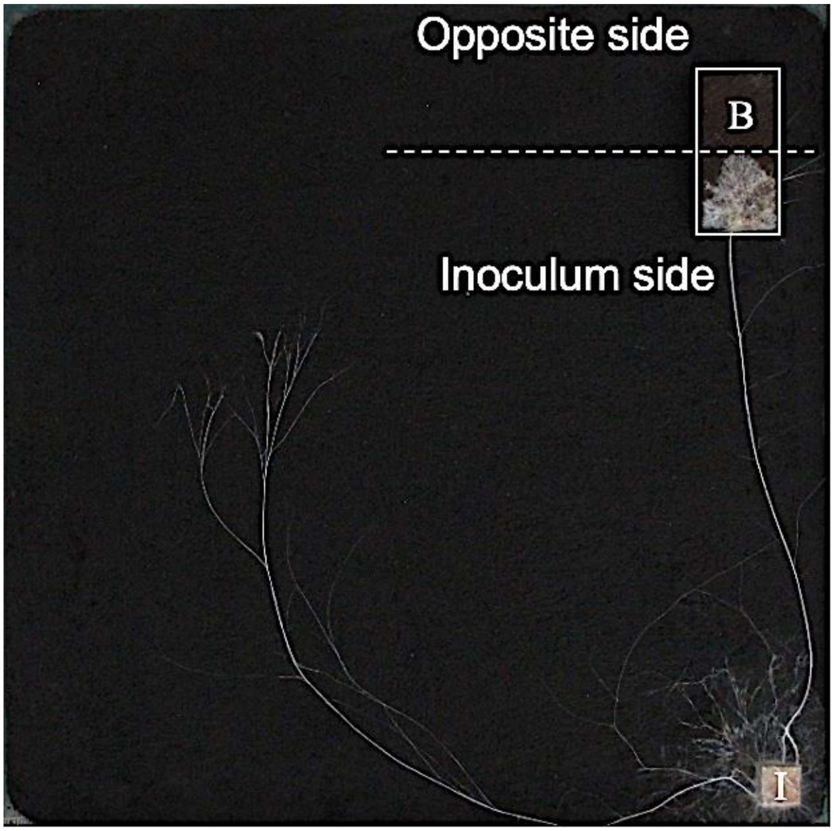

Supplementary Figure 2. Diagram showing definition of inoculum side and opposite side of a bait wood block (B). Each image of bait wood block was split into two parts at the center line. “I” indicates inoculum. The outline of the bait wood block is delineated by a white line for clarity. Colonization of mycelium occurred from inoculum side in this case.

3 Supplementary Figure 3

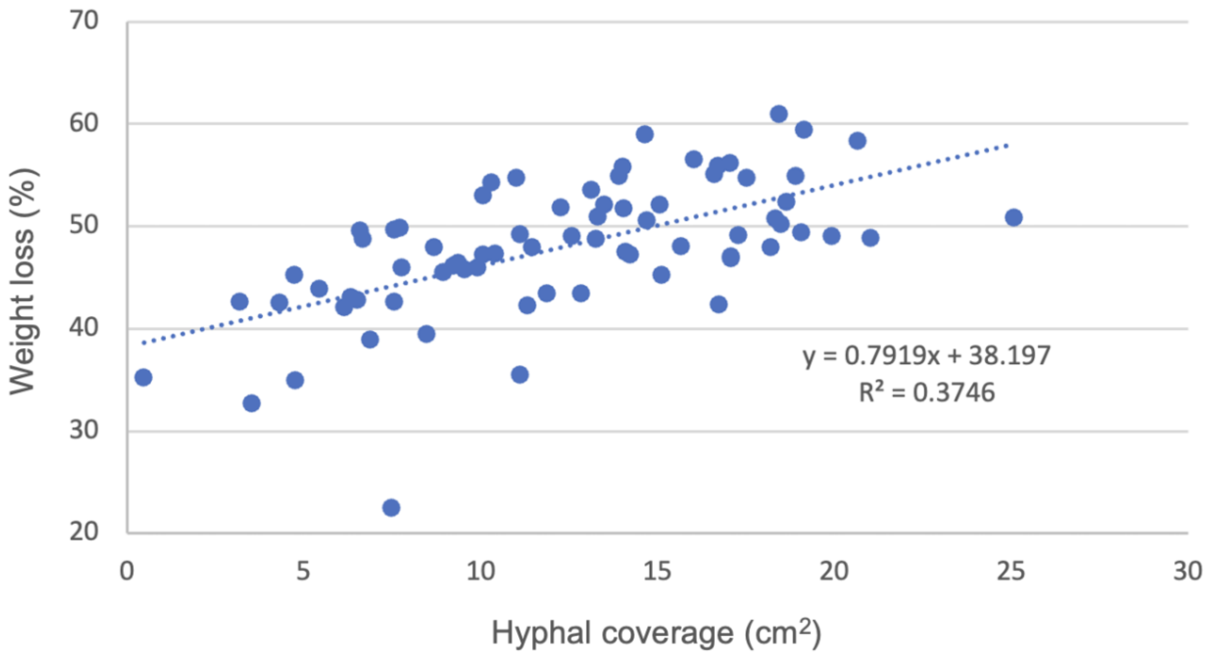

Supplementary Figure 3. The regression line obtained from the data of hyphal coverage on soil and weight loss of 73 inoculum wood blocks, which were not used for the incubation period I and II, after the preincubation period.
